# Crowding Effects during DNA Translocation in Nanopipettes

**DOI:** 10.1021/acsnano.5c01529

**Published:** 2025-04-23

**Authors:** Rand A. Al-Waqfi, Cengiz J. Khan, Oliver J. Irving, Lauren Matthews, Tim Albrecht

**Affiliations:** † University of Birmingham, School of Chemistry, Edgbaston Campus, Birmingham B15 2TT, U.K.; ‡ Department of Medicinal Chemistry and Pharmacognosy, Faculty of Pharmacy, Jordan University of Science and Technology, P.O. Box 3030, Irbid 22110, Jordan; § Federal Institute for Materials Research and Testing, Department 6, Unter den Eichen 87, 12205 Berlin, Germany

**Keywords:** DNA translocation, transport, resistive-pulse sensing, nanopores, nanopipettes, crowding, confinement

## Abstract

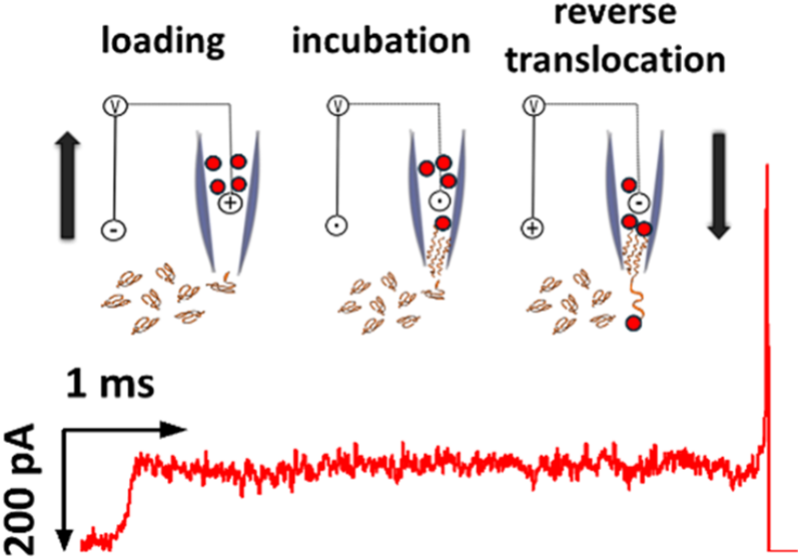

Quartz nanopipettes are an important emerging class of electric single-molecule sensors for DNA, proteins, their complexes, as well as other biomolecular targets. However, in comparison to other resistive pulse sensors, nanopipettes constitute a highly asymmetric environment and the transport of ions and biopolymers can become strongly direction-dependent. For double-stranded DNA, this can include the characteristic translocation time and tertiary structure, but as we show here, nanoconfinement can also unlock capabilities for biophysical and bioanalytical studies at the single-molecule level. To this end, we show how the accumulation of DNA inside the nanochannel leads to crowding effects, and in some cases reversible blocking of DNA entry, and provide a detailed analysis based on a range of different DNA samples and experimental conditions. Moreover, using biotin-functionalized DNA and streptavidin-modified gold nanoparticles as target, we demonstrate in a proof-of-concept study how the crowding effect, and the resulting increased residence time in nanochannel, can be exploited by first injecting the DNA into the nanochannel, followed by incubation with the nanoparticle target and analysis of the complex by reverse translocation. We thereby integrate elements of sample processing and detection into the nanopipette, as an important conceptual advance, and make a case for the wider applicability of this device concept.

Resistive-pulse sensors are an important class of single-molecule sensors and broadly fall into three classes, namely biological, solid-state or silicon chip-based nanopores, and nanopipettes.^1−10^ They share certain common features, for example, that typically a single channel connects two liquid compartments, which are otherwise separated through a highly insulating membrane. The transport of individual biomolecular analytes through the channel (“translocation”) typically alters the resistance of the channel temporarily, resulting in a measurable, transient change in the electric current through the system.^1^

In biological nanopore sensors, the channel is often constituted by a pore-forming protein, such as α-hemolysin, embedded in a lipid bilayer membrane. Very small pores can be fabricated with high precision in this way, which is why such pores are being used for DNA and, more recently, explored toward protein sequencing.^11^ In chip-based nanopore sensors, pores are formed by electron or ion beam milling into a thin, solid-state membrane, for example made of silicon nitride, graphene, or another 2D material, or by using strong, localized electric fields.^12,13^ More detailed reviews of these two classes can be found elsewhere.^13−15^ Notably, however, in both of these, the transport of ions or biomolecular analytes between the bulk of the solution and the pore channel is comparable on both sides of the membrane. Accordingly, “capture and recapture” experiments, where an analyte is first translocated in one direction and then recaptured by a fast bias reversal, yielded similar results for both translocation directions, for example, in terms of the observed average dwell times for double-stranded (ds) DNA and nanoparticles.^16−18^

On the other hand, nanopipette-based sensors are fabricated from (quartz) capillaries using a pipet puller, i.e., involving a combination of localized laser-induced heating and mechanical pulling. The pore channel is usually narrowest at the tip of the pipet, with inner diameters typically in the range of 5–20 nm, and then gradually widens up to the diameter of the capillary.^4,19^ Hence, opening angles smaller than 10° and taper lengths of several mm are typical, while the sensing region is still confined to the narrowest part of the channel (say, the first 50 nm taken from the end of the tip).^20^ As a result, compared to the outside of the pipet, transport on the inside is geometrically restricted, the electric field decays more gradually from the tip, and surface effects are more prominent.^4^ In the past, this has been exploited for the localization and controlled delivery of both single-stranded (ss) and dsDNA^21,22^ and more recently sparked renewed interest in understanding and exploiting the said asymmetry effects in the translocation of linear and functionalized dsDNA.^23−26^

Here, we provide a systematic analysis of dsDNA translocation in both directions, i.e., from the outside to the inside (“in”) and the inside to the outside of the pipet (“out”), for a range of different DNA lengths from 4 to 48.5 kbp and at different bias voltages, Figure 1. Importantly, and in contrast to previous literature, nanopipettes were loaded with 100s of dsDNA molecules for extended periods of time before initiating reverse translocation. This allows for the investigation of distinct features of the nanopipette sensor, such as crowding effects and the emergence of a new, distinct “translocation state” of the nanopipette once the number of translocated DNA molecules passes a certain DNA length-dependent threshold value. This state is characterized by an increased translocation time and broadening of the translocation time distribution, however, without a significant change in either pore conductance or electric noise. In the case of 10 and 48.5 kbp λ-DNA, after continued operation, translocation events were eventually no longer detected, while the pore conductance decreased (albeit not to zero) and the electrical noise increased. This effect was found to be reversible. Finally, we show that the temporary storage of functionalized DNA in the nanochannel can be exploited for incubation, target capture, and read-out by reverse translocation. This is demonstrated in a proof-of-concept study for nanometer-sized, streptavidin-modified Au particles, but clearly opens up avenues for integrated sample processing and read-out for other target analytes, such as biomarker proteins or RNA.

**Figure 1:**
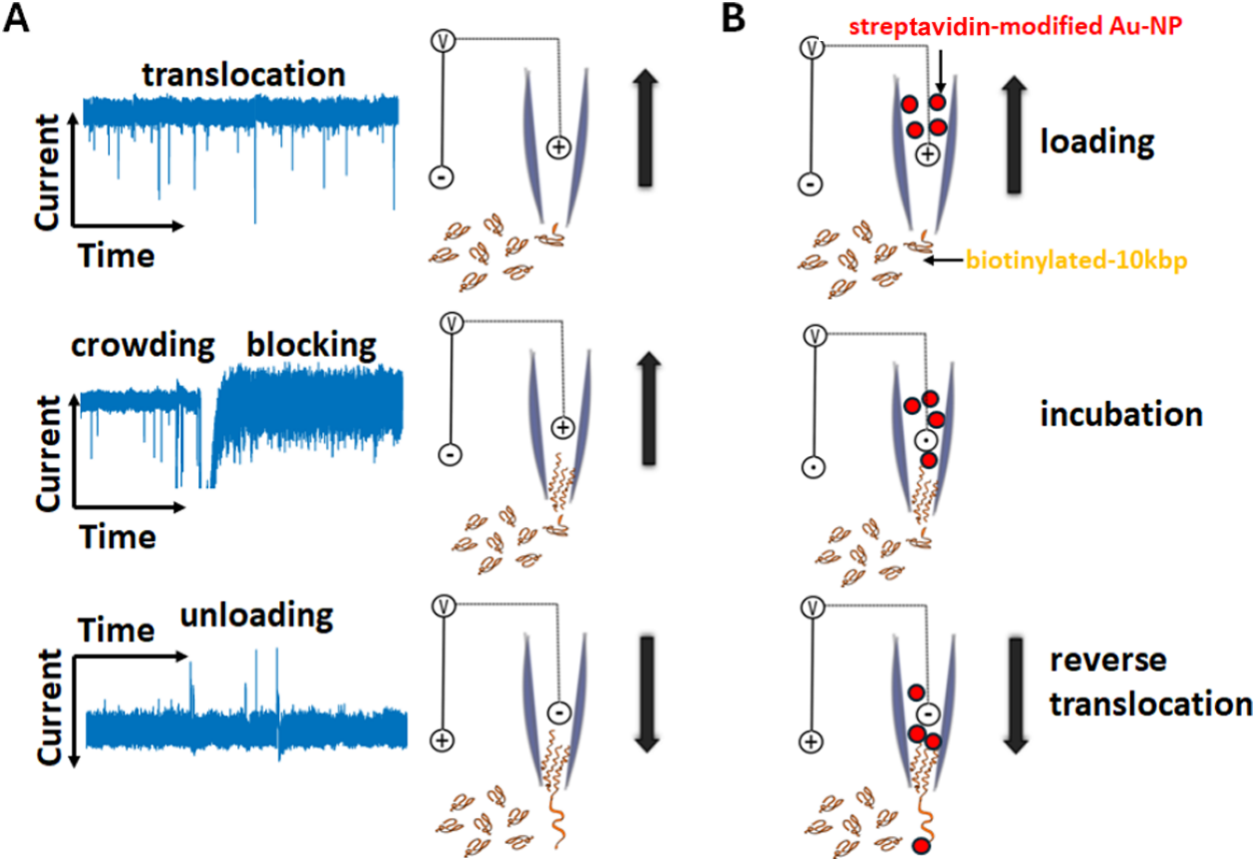
Illustration of two experimental paradigms described in this work. (A) Translocation of long kbp DNA into the nanopipette (top), crowding of the nanochannel and eventual blocking of DNA translocation (center), and, last, unloading of the nanopipette upon bias reversal (bottom). (B) Capture of streptavidin-modified nanoparticles with functionalized (biotinylated) dsDNA after loading, incubation, and reverse translocation for detection and analysis.

## Results and Discussion

**Figure 2:**
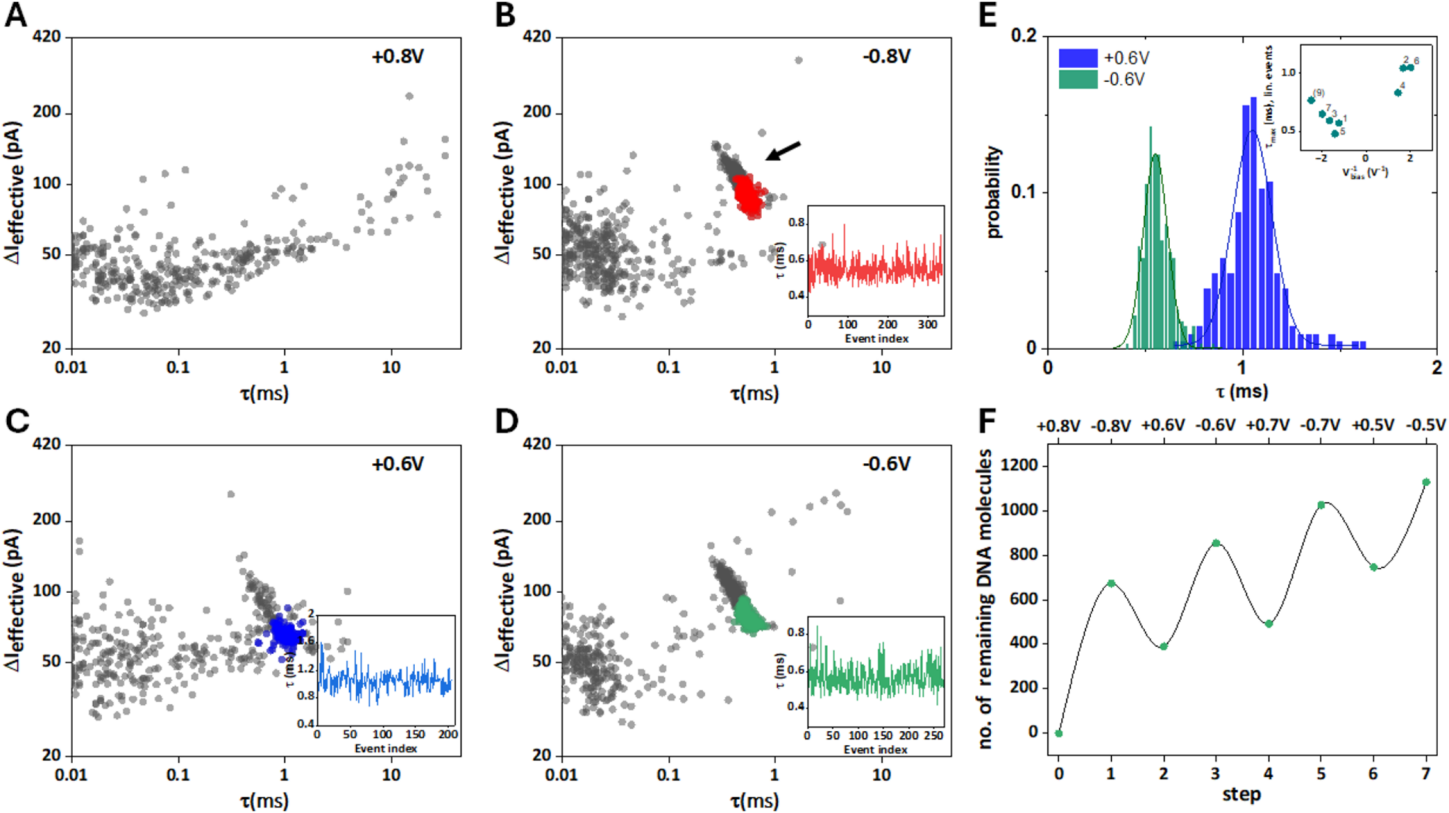
Translocation of 4 kbp DNA. (A–D) Scatter plots of event magnitude vs duration for steps 0–3 (*V*_bias_ as indicated). Step 0 shows no DNA translocation, as there is no DNA present inside the nanopipette initially. Event clusters occur in subsequent steps (see arrow in panel B), and linear events are color-coded, as discussed in the main text. Insets: event duration vs event index (linear events only). (E) Translocation time distributions for linear events only, step 2 (blue) and step 3 (green), including normal distribution fits. Inset: a plot of τ_max_ vs *V*_bias_^–1^ appears to be linear (steps 0–7 and 9), consistent with electrophoretically driven transport. The signal-to-noise ratio in step 8 (*V*_bias_ = +0.4 V) was low and this data set was therefore excluded from further analysis. (F) Number of DNA molecules inside the nanopipette, based on translocation statistics for steps 0–7.

As a first step, we investigated the translocation characteristics of 4 kbp DNA in 4 M LiCl, and in particular their dependence on the direction of transport into or out of the nanopipette. The results for the first four steps in the bias sequence, namely +0.8/–0.8/+0.6/–0.6 V, are summarized in Figure 2 (*c*_DNA,out_ = 300 pM, initially *c*_DNA,in_ = 0). Recording of the current–time data, event detection, and analysis are described in the Methods Section. Example traces are shown in section 3 of the Supporting Information.

Panels A-D show scatter plots of the effective event current magnitude, Δ*I*_eff_, vs the dwell time τ. For *V*_bias_ = +0.8 V (panel A, step 0), given the bias polarity, no DNA translocation is expected, as initially *c*_DNA,in_ = 0, and hence no DNA event cluster is observed.^5,27,28^ Subsequently, *V*_bias_ is switched to −0.8 V (B, step 1) and DNA translocates from the outside to the inside of the nanopipette. Accordingly, a distinct cluster of DNA translocation events appears at approximately 1 ms dwell time and an event magnitude of 100 pA (as indicated by the arrow; 673 events, average translocation frequency *f*_1_ = 0.75 s^–1^). Within this cluster, events at the top left are due to the translocation of folded DNA (and are thus shorter and more intense), while events at the bottom right more likely result from the translocation of DNA in a linear configuration (highlighted in red; cf. Section S4).^5,20^ We formally identified 370 events as linear and 303 events as folded (linear/folded ratio = 0.55), which is in line with previous literature for nanopores of similar size.^19,29^ A plot of τ vs the event index for those linear events shows no apparent correlation (inset), as expected for a stochastic process of this kind.

When *V*_bias_ is changed to +0.6 V (C, step 2), some of the DNA that had previously entered the pipet in step 1 now translocates from the inside of the pipet to the outside (283 DNA events detected, *f*_2_ = 0.3 s^–1^). Linear events in the event cluster are highlighted in blue (linear/folded ratio = 0.77) and for those, there was again no apparent correlation between τ and event index. The series continues with *V*_bias_ = −0.6 V (D, step 3), where 464 DNA events were detected (linear events in green, f_3_ = 0.5 s^–1^, linear/folded ratio = 0.59), and subsequently *V*_bias_ = ± 0.7 V (steps 4 + 5) and *V*_bias_ = ± 0.5 V (steps 6 + 7), see further details in the Section S8. Note that each *V*_bias_ value is maintained for 1000 s, so the average residence time of the DNA inside the nanopipette is longer than in experiments where the DNA is translocated and then rapidly recaptured.^24−26^ A few observations are appropriate at this stage. First, there was a moderate effect of the translocation direction on the linear/folded ratio, which was on average approximately 30% higher for translocation from the inside to the outside of the pipet. Second, the translocation time distribution was shifted to longer times but had a similar relative standard deviation, σ/τ_max_ ≈ 0.1, cf., panel E. This is further illustrated by a plot of the most probable translocation time τ_max_ vs *V*_bias_^–1^, which shows a consistent upward shift of the positive branch, relative to the negative one, panel E inset. Furthermore, a linear correlation between τ_max_ and *V*_bias_^–1^ is consistent with electrophoretically driven transport of DNA.^1^ Third, the translocation frequencies f for the unloading steps 2, 4, and 6 are similar in magnitude, compared to the loading steps 1, 3, 5, and 7, even though the average concentrations of DNA on the outside and the inside of the pipet are expected to be very different. Taking steps 1 + 2 as an example, while *c*_DNA,out_ = 300 pM, *c*_DNA,in_ is given by the number of DNA molecules that translocated into the pipet in step 1 (673) and volume of electrolyte solution inside the pipet (∼7 μL). This yields approximately, *c*_DNA,in_ ≈ 0.2 fM. Ignoring differences in capture geometry and electric field distribution on the inside and the outside of the pipet for the time being, in electrophoretically dominated transport f ∝ *c*_DNA_ and therefore the expected translocation frequency for the unloading steps should be about 10^6^–10^7^ times smaller than for the loading steps. This is not the case, which suggests that the solution on the inside of the pipet, namely in the confined region, is not well mixed and that the local DNA concentration close to the nanopipette tip is increased.

Since the number of translocated DNA molecules is known for each step, the number of DNA molecules remaining inside the pipet can be estimated. The result of this analysis is depicted in panel F, which, apart from oscillations due to consecutive loading/unloading cycles, shows a steady increase over the course of the experiment. As shown below, this is qualitatively different for the longer DNA samples studied here.

**Figure 3:**
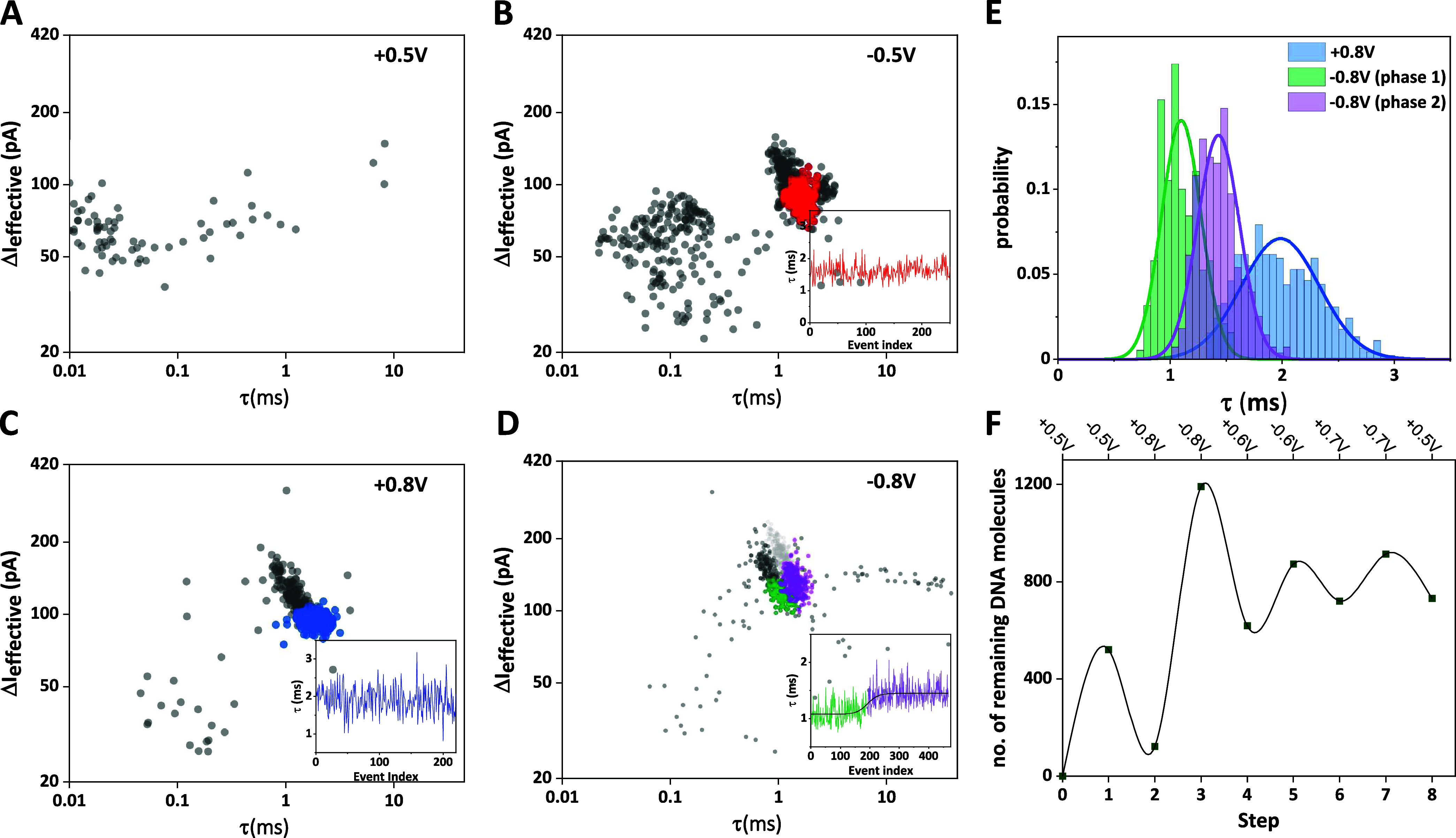
Translocation of 7 kbp DNA. (A–D) Scatter plots of Δ*I*_eff_ vs τ for steps 0–3 (*V*_bias_ as indicated). Step 0 shows no DNA translocation, as there is initially no DNA present inside the nanopipette. Event clusters occur in subsequent steps, linear events are color-coded, as discussed in the main text. Insets: τ vs event index (linear events only). (E) τ distributions for linear events only, step 2 (blue) and step 3, phase 1 (green) and phase 2 (magenta), including normal distribution fits. (F) Number of DNA molecules inside the nanopipette, based on translocation statistics, for each step.

In the case of 7 kbp DNA, a similar experimental design was followed, albeit with a bias sequence of ±0.5 V; ±0.8 V; ±0.6 V; ±0.7 V and a final step at +0.5 V (*c*_DNA,out_ = 600 pM), Figure 3. Accordingly, no DNA translocation events are detected in step 0, since *c*_DNA,in_ = 0 (panel A, *V*_bias_ = +0.5 V). In step 1 (*V*_bias_ = −0.5 V, panel B), DNA translocates into the nanopipette and an event cluster with approximately 1 ms duration and 100 pA event magnitude is detected. Again, translocation events classified as linear are highlighted in red, the τ vs event index plot is shown in the inset. No apparent correlation between the latter two parameters was observed for this subset of events. Interestingly, however, in the scatter plot, there appears to be a tail of events with longer durations and magnitudes similar to linear DNA events, which was absent in the data obtained for 4 kbp DNA, cf. Figure 2B.

Unloading of the pipet occurs in step 2 (*V*_bias_ = +0.8 V, panel C), where translocation events form a distinct cluster. Linear events are highlighted in blue and the τ vs event index plot (inset) again shows no apparent correlation. Surprisingly, in step 3 (*V*_bias_ = −0.8 V, panel D), translocation occurs in different phases. In phase 1, a cluster of DNA-related events emerges at shorter τ (linear and folded events in green and dark gray, respectively), while no correlation is apparent in the τ vs event index plot (inset, green section). However, as DNA continues to translocate into the nanopipette, τ not only gradually increases, but seems to transition to a new steady-state value (inset, magenta section). Accordingly, in the cluster plot, the event cluster is nominally shifted to longer τ at comparable ΔI_eff_ (linear and folded events in magenta and light gray), suggesting that subsequent DNA requires more time to translocate. This could be due to a decrease in the local electric field, but given that the pore current does not change significantly, we consider this unlikely. However, in line with our discussion above, namely that the local concentration of DNA in the pipet tip may be enhanced, we hypothesize that the slowing of the translocation process could be due to increased friction inside the nanochannel, i.e., between the incoming DNA strand and those already present. Notably, we consistently make similar observations with other, longer DNA samples in this study, an aspect we will return to below.

Further analysis of the τ distribution for steps 2 (blue) and 3 (green/magenta) is shown in panel E, including Gaussian fits. In step 2, the τ distribution is shifted toward longer times and is broader (τ_max_ = 2.0 ms; σ/τ_max_ = 0.18), while for step 3, τ_max,1_ = 1.1 ms (σ_1_/τ_max,1_ = 0.16) in phase 1, and τ_max,2_ = 1.4 ms (σ_2_/τ_max,2_ = 0.13) in phase 2. Thus, despite the differences in position and width, the relative standard deviation, σ_1_/τ_max_, is comparable in all three cases.

Finally, the number of DNA molecules remaining in the nanopipette during each step is shown in panel F. This number initially increases (steps 0–3), similar to what has been observed for the 4 kbp DNA above, but then seems to be oscillating around a plateau from step 3 onward. Hence, while DNA is still transported into and out of the nanopipette, the uptake capacity seems to be limited, consistent with the above hypothesis that DNA remains trapped in the nanochannel. Interestingly, we observed the same qualitative behavior, as well as some intriguing differences, for 10 kbp and 48.5 kbp DNA.

**Figure 4:**
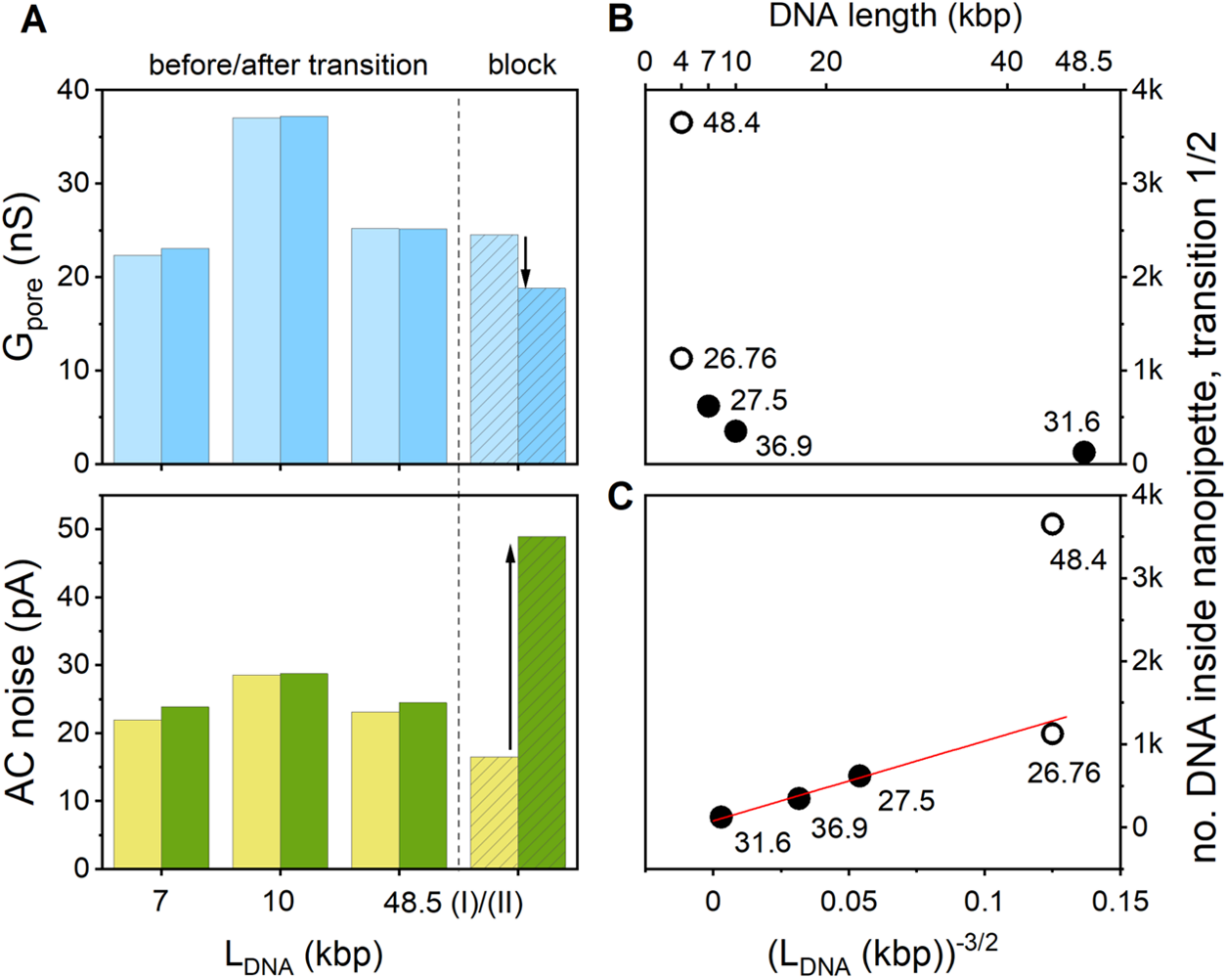
(A) *G*_pore_ (DC channel, top) and electric (AC channel, bottom) before and after the transition from phase 1 to phase 2, left/light blue and right/dark blue, for 7, 10, and 48.5 kbp DNA (“48.5 (I)). “Block” (“48.5 (II)”) refers to a second transition observed for 48.5 kbp DNA, see main text. (B) Plot of the no. of DNA molecules in the nanopipette at the end of the bias sequence (4 kbp DNA) or until the transition point from phase 1 to phase 2 is reached (7, 10, and 48.5 kbp DNA). The labels indicate *G*_pore_ of the nanopipette (in nS), as determined by I/V spectroscopy prior to the experiment. (C) Plot of the number of DNA molecules inside the nanopipette at the transition from phase 1 to 2 vs . A linear relationship is consistent with a space-limiting model of translocation into the nanopipette, as discussed in the main text.

Before exploring the latter aspects, we further analyzed other electric characteristics of the sensor before and after the transition from phase 1 to phase 2, specifically the pore conductance G_pore_ and the electric noise from the “DC” and “AC” channels of the setup, respectively (see Methodology). In this context, it is worth reiterating that, due to the design of our amplifier with two output channels, the “DC” channel effectively contains the steady-state (open) pore current, hence *G*_pore_ ≈ *I*_DC_/*V*_bias_, while the “AC” channel records fast modulations of the current, including typical translocation events. Accordingly, in Figure 4A, for the different DNA lengths used in this study, we show *G*_pore_ before (left) and after (right) the transition between different phases (top) as well as the standard deviation of the current in the “AC” channel (bottom). This includes transitions from phase 1 to 2 and, for 48.5 kbp DNA, also the transition to a third phase, as discussed below. Focusing on the transition from phase 1 to 2 initially, it is notable that despite the emergence of a second translocation cluster, cf. Figure 3D, no significant change in either *G*_pore_ or AC noise was observed. With the nanopore current largely unchanged, it is thus unlikely that the slowing of the translocation process in phase 2 is due to a change in the local electric field distribution and more likely to do with friction effects, as suggested above. For 48.5 kbp DNA, we further observed a second transition (“48.5 (II)” in panel A), where DNA translocation was no longer observed (“block”). This was accompanied by a significant (23%) drop in *G*_pore_ and a tripling of the AC noise, indicative of a significant change in the sensing region of the nanopipette.^30^ We return to a more detailed analysis below. Notably, however, *G*_pore_ does not drop to zero, which could mean that the remaining pore current is due to continued ion transport through a DNA-rich region in the tip, leakage current through the thin quartz walls,^31^ or indeed a combination of both.

Taken together, however, it seemed that for longer DNA the transition from phase 1 to 2 and potentially to phase 3, where applicable, was reached sooner than for shorter DNA. This led us to investigate in a more systematic manner the relationship between the DNA length, the number of DNA molecules resident in the nanopipette at the 1:2 transition and G_pore_. Hence, panel B shows a plot of the total number of DNA molecules in the nanopipette up to the end of the bias sequence (4 kbp DNA, open circles) and to the transition from phase 1 to 2 (7, 10, and 48.5 kbp DNA (solid circles)). The numeric label associated with each data point is the value of *G*_pore_ (in nS), as a proxy for the nanopipette size (see Methods Section and Section S6).

Taking the two results for the 4 kbp DNA sample first, it is apparent that after the same duration, more DNA molecules have translocated into the larger nanopipette (48.4 nS), compared to the smaller one (26.76 nS), as expected in the transport-limited regime.^32^ The transition to phase 2 had not occurred in either of the two cases under the experimental conditions used.

For the longer DNA samples in nanopipettes with comparable *G*_pore_, the number of DNA molecules required to reach the 1/2 transition indeed decreases with length, from 619 for 7 kbp to 349 for 10 kbp and 124 for 48.5 kbp. This observation may be rationalized based on a simple model. To a first approximation, we assume that the nanochannel close to the pipet tip is characterized by an effective, finite volume, *V*_ch_, that may be filled by DNA with a molecular volume *V*_DNA_. Treating the DNA as a worm-like chain and , the number of DNA in *V*_ch_ is then simply , where *R*_H_ is the hydrodynamic radius, P is the persistence length, *N*_bp_ is the number of base pairs, and *d*_bp_ is the average distance between two successive ones, see Section S7 for further details. This expression predicts a linear correlation between *N*_DNA_ and  with slope , which is indeed consistent with the results displayed in panel C. A linear least-squares fit yields a slope of (3.0 ± 0.4)·10^8^ bp^3/2^/nm^3^ (intercept: 80 ± 40). Taking *P* = 50 nm and *d*_bp_ = 0.34 nm, this provides *V*_ch_ ≈ 5.1·10^9^ nm^3^ or 5.1 μm^3^. Approximating the nanochannel tip as a cone with base radius *r*, height *h*, and opening angle 2α, its volume becomes . Setting *V*_ch_ ≈ *V*_cone_, α ≈ 5°, and solving for *h* yields 8.6 μm (*r* ≈ 0.75 μm), suggesting that the extension of the nanoconfined region may be significantly smaller than the taper length of the pipet (≈ 3 mm, see Table S1).

Extrapolation toward 4 kbp DNA suggests that, for similar *G*_pore_, the critical limit of ≈1300 molecules was not reached under the experimental conditions used here, and therefore, no 1:2 transition was observed. Hence, while the model is relatively simple, it seems to capture some essential features in the experimental data. In future, it could be refined further, e.g., by more accurately describing the DNA polymer in confinement, and complemented with systematic experimental studies, e.g., involving nanopipettes with different shapes and geometries in the nanochannel.

**Figure 5:**
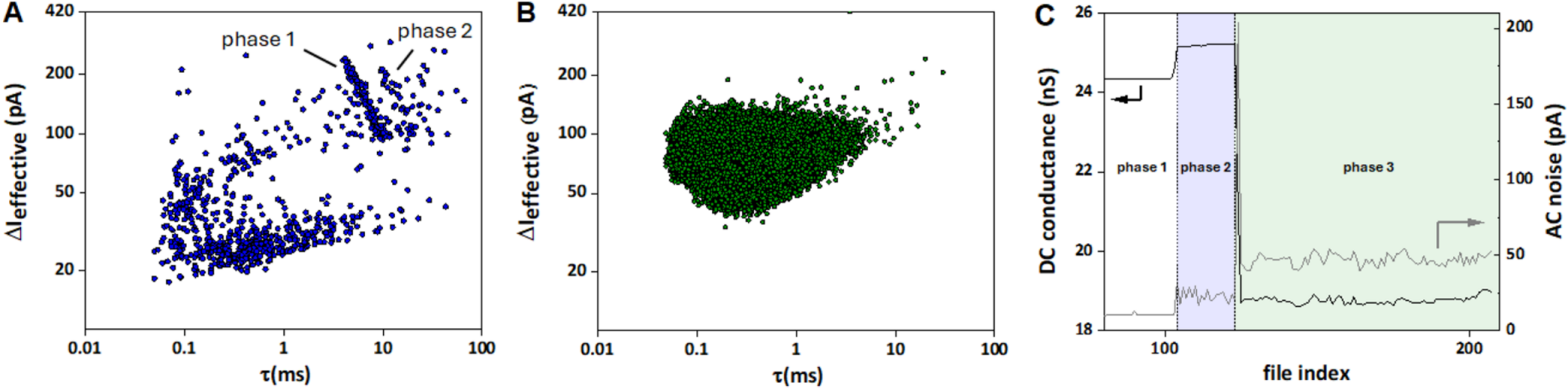
(A, B) Scatter plots of ΔI_e_ vs τ for phases 1 + 2 (A) and phase 3 (B) of a translocation experiment with 48.5 kbp λ-DNA (*V*_bias_ = −0.5 V, event detection threshold: 3σ). (C) *G*_pore_ and the current noise in the AC channel, as a function of file index (1 file = 10 s run time). Phase 2 is highlighted in blue and corresponds to the emergence of a second cluster of translocation events (panel A). In the “blocking” phase (green), no further translocation events could be detected, as shown in panel B.

However, here we return to a more detailed analysis of the translocation data for 48.5 kbp DNA, Figure 5 (*V*_bias_ = −0.5 V, *c*_DNA,out_ = 300 pM). As shown in panel A, DNA translocation initially results in a well-defined cluster of events at τ ≈ 4–11 ms and Δ*I*_e_ ≈ 100–200 pA (cf. “phase 1” in panel C). The end of phase 1 is marked by a small increase in *G*_pore_ of ≈0.8 nS (3%) and an approximate doubling of the noise in the AC channel from about 10 to 22 pA. Subsequently, a second cluster of translocation events began to emerge at τ ≈ 10–20 ms and similar Δ*I*_e_, while *G*_pore_ and AC noise remained approximately constant (cf. “phase 2” in panel C). The end of this phase is marked by a significant (23%) decrease of *G*_pore_ (cf. “48.5 (II)” in Figure 4A), an initial spike, and then a new steady-state value of the AC channel noise of ≈50 pA (“phase 3”, panel C). During this third phase, no further DNA translocation could be detected over 800 s recording time, as shown in the scatter plot in panel B. Taken together, it appears that DNA initially translocates in an uninhibited fashion (phase 1), then enters a phase where the speed of DNA translocation is reduced, most likely to DNA crowding inside the pipet tip (phase 2) and finally translocation eventually ceases when the nanochannel becomes too densely populated to accommodate more DNA molecules (phase 3). Notably, this effect was reversible in that, when the bias was reversed, DNA was transported out of the nanopipette and the original *G*_pore_ and AC noise values were recovered (see Figure S10, for data with 10 kbp DNA).

When decreasing the DNA concentration outside the nanopipette from *c*_DNA,out_ = 300 pM to 40 pM (*G*_pore_ = 44.1 nS), translocation experiments over the same time duration did not lead to the same blocking behavior, cf. Figure S8 in the SI. In light of the above discussion, this is unsurprising: the lower concentration leads to a significant reduction in the translocation frequency for transport into nanopipette and even if transport within the nanochannel is restricted, the number of resident DNA molecules does not reach the critical value.

The results presented so far provide compelling evidence for the prolonged presence of DNA inside the nanochannel and its effect on the behavior and operational characteristics of the nanopipette during resistive-pulse sensing experiments. The combination of reversible loading, local storage and unloading of the DNA, however, raises interesting prospects for sensor applications. For example, DNA functionalized with appropriate capture probes (“carrier DNA”) could initially be kept outside the nanopipette, while their biomolecular targets are largely confined to the inside. The DNA could then be translocated inward, incubated with the target in the confined region for a suitable amount of time, and finally translocated in the reverse direction to detect where and how many targets are bound to the carrier. The benefits include not only the smaller sample volume on the inside of the pipet and potentially operation in asymmetric electrolyte conditions, but also take advantage of the somewhat increased linear-to-folded ratio for DNA translocation in this direction (which simplifies readout of the capture probe locations), vide supra. We demonstrate this approach in a proof-of-concept experiment below. In this instance, we chose gold nanoparticles as targets, as their dispersion in the translocation buffer was found to be sufficiently stable and their relatively large size (compared to some protein targets, for example) rendered them easily detectable.

Specifically, for the carrier we prepared 10 kbp DNA functionalized with a biotin group at one end, cf. Section S1, and added it to the electrolyte solution (4 M LiCl) on the outside of the pipet. The solution on the inside of the pipet contained streptavidin-functionalized Au particles in the same electrolyte (Nanopartz Inc.; diameter: 40 nm, *c*_particle,in_ = 500 pM), but initially no DNA. We then translocated DNA into the pipet for 1000 s (*V*_bias_ = −0.7 V; *G*_pore_ = 26.1 nS), switched the applied voltage bias off and left the sample to incubate for ∼20 min. Subsequently, the bias was reversed for 1000 s (*V*_bias_ = +0.7 V) and the DNA/particle mixture translocated from the inside to the outside of the pipet (“reverse translocation”). Our expectation was that, due to the location of the biotin capture probe, translocation events of the DNA/nanoparticle complex would feature nanoparticle-related subevents either at the beginning or at the end of a translocation event (depending on in which direction the DNA enters the pore),^33^ thereby confirming the successful binding and detection of the target analyte.

**Figure 6:**
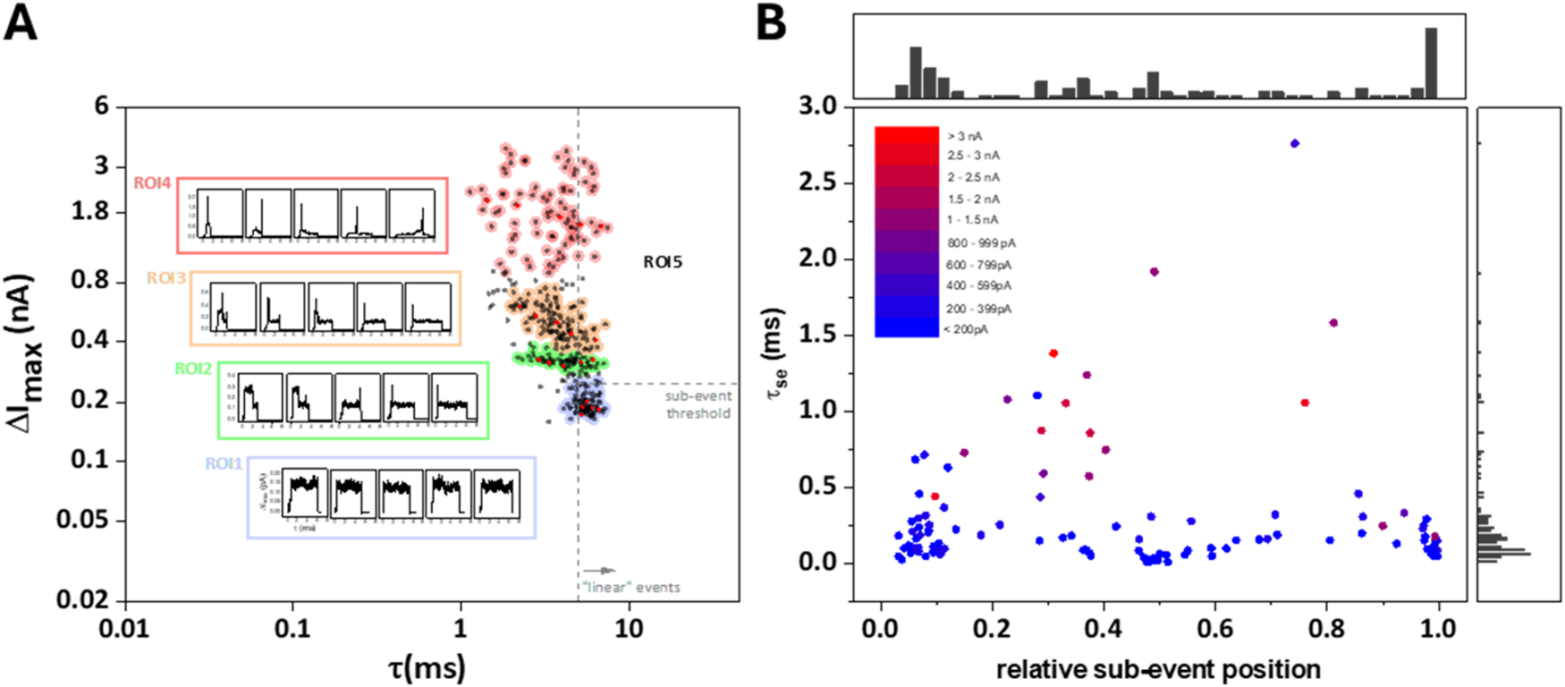
Reverse translocation of biotin-functionalized, 10 kbp DNA and 40 nm streptavidin-modified nanoparticles (4 M LiCl electrolyte, *G*_pore_ = 26.1 nS; *V*_bias_ = +0.7 V). (A) Scatter plot of Δ*I*_max_ vs τ. Regions-of-Interest (RoI) 1–5 are indicated with representative examples (inset), see main text for further discussion. (B) For events in RoI5, subevent duration τ_se_ vs relative subevent position within an event (0: event start, 1: event end). Color code: subevent magnitude, from <200 pA (blue) to >3 nA (red). Top and right-hand side: histograms of the relative subevent position and subevent duration.

This is indeed borne out in the results shown in Figure 6. Specifically, panel A shows a scatter plot of the maximum event current Δ*I*_max_ vs τ. To this end, Δ*I*_max_ was chosen over Δ*I*_eff_, in order to emphasize the difference between events with and without subevents. Background noise-related events have been removed for clarity.

Not unexpectedly, the Δ*I*_max_ vs τ plot is more complex than those obtained from the translocation of unmodified DNA, cf. Figures 2 and 3. Thus, as a first step, we explored the data set through manually selected Regions-of-Interest (RoI), as indicated, with the aim of a high-level classification of event types. Randomly selected example events are shown in the insets with corresponding data points in red (from left to right). Starting with RoI1 (light blue), these events occur at relatively long τ and low Δ*I*_max_, have no significant substructure and are predominantly due to the translocation of linear DNA. RoI2 (green) is characterized by a cluster of events with a relatively wide τ- but remarkably narrow Δ*I*_max_ distributions, with Δ*I*_max_ values ranging from approximately 300–400 pA. Closer inspection of the event shapes indicates that events at longer τ tend to be linear DNA translocation events with short, spike-like subevents. Those at shorter τ display the characteristic features of folded DNA translocation, where the beginning of an event is characterized by an excursion to approximately twice the Δ*I*_max_ value for linear DNA translocation with a significant duration, τ_se_ (see the two example events on the left-hand side of RoI2). Event shapes in RoI3 (orange) again provide evidence for linear and folded DNA translocation (from longer to shorter τ values, right to left), but the event magnitudes are typically defined by short, intensive subevents (Δ*I*_max_ < 0.8 nA). Finally, RoI4 (light red) encompasses events in a more diffuse cluster that are of similar appearance to those in RoI3, albeit with even larger subevents (Δ*I*_max_ > 1 nA). This event magnitude is indeed comparable to the current change observed when the nanopipettes are seemingly blocked with DNA, cf. Figure 5, and we therefore speculate that events in RoI4 may involve pipet blockage with sufficiently large nanoparticles.

However, returning to the original hypothesis, namely whether we can confirm nanoparticle binding to the functionalized (biotinylated) part of the DNA, we chose to define a further RoI, RoI5. To this end, we determined the mean τ_m,1_ and standard deviation σ_m,1_ from a Gaussian fit of the translocation time distribution for the events in RoI1 (linear events only; τ_m,1_ = 0.0057 ms, σ_m,1_ = 0.0007 ms) and then considered all events with τ > (τ_m,1_ – σ_m,1_) and Δ*I*_max_ > 250 pA as nominally linear DNA translocation events with subevents. In this way, we identified 63 events from RoI1–4, which were then subjected to a subevent search. This identified 115 subevents in total, namely 34 events with one subevent, 17 with two, 9 with three, and two with four. One event with one significant subevent feature as well as two others were misclassified; a broad selection of individual events is shown in the Section S10 for reference. Figure 6B shows the results of this analysis as a plot of τ_se_ vs the relative subevent position within an event (0: event start, 1: event end), along with the respective histograms at the top and the right-hand side. The color code represents the subevent intensity, from <200 pA (blue) to >3 nA (red). From this analysis, it becomes apparent that the majority of subevents occurs close to the start or the end of an event (66 out of 115 within the first or last 15% of the event), that the dominant τ_se_ is relatively short (mode = 60 μs, see τ_se_ histogram), and their magnitude low (<400 pA). Hence, this statistical distribution of subevents indeed provides evidence that the nanoparticles are bound to the DNA and detected successfully in this experimental configuration. Interestingly, there also appears to be a somewhat increased probability of low-magnitude, short subevents close to the central region of translocation events (relative position ≈0.5), even though there is no obvious streptavidin binding site in this part of the functionalized DNA. While statistically the simultaneous translocation of DNA and nanoparticles (“co-translocation”) is not unexpected, it is not immediately obvious why nanoparticles should more likely cotranslocate in any particular region of an event and should be more evenly distributed. Possible reasons could be related to specific DNA/nanoparticle interactions or the dynamics of the translocation process, but this aspect requires further study.

## Conclusions

In conclusion, we have shown through a range of experiments how confinement in the nanopipette can influence the translocation of kbp DNA into and out of the nanopipette tip. Slow transport from the tip region into the bulk of the nanopipette, likely due to a combination of spatial constraints, friction, and weakening electric field, appears to be important in this context. Specifically, after loading the nanopipettes with DNA, the translocation frequency for reverse translocation (out of the pipet) was much higher than expected, based on estimates for homogeneous mixing, and compared to inward translocation (i.e., the local concentration in the tip was enhanced). In line with previous studies, we found inward translocation to be faster than outward translocation, while the variance of the translocation time distribution scaled accordingly. We also observed a somewhat larger fraction of linear DNA when translocating out of the pipet, for all DNA lengths studied here, which again may be related to confinement inside the nanopipette tip.

To this end, confinement effects were particularly prominent for longer DNA, where we observed that the translocation process undergoes different stages, depending on the number of DNA molecules resident in the pipet. While initially all DNA samples displayed unperturbed translocation into the pipet (“open” state), with well-defined translocation time distributions for the translocation of linear and folded DNA, the system was found to switch to a new translocation state once a critical number of DNA inside the pipet—and by implication, inside the nanochannel—was reached (“crowded” state). This limiting value was smaller for longer DNA for comparable nanopipette sizes and we have presented evidence that this is consistent with a “finite volume” model of the tip region. While in our study all nanopipettes were fabricated with similar pulling parameters and displayed comparable *G*_pore_ values (with the exception of one data set for 4 kbp DNA, vide supra), we envisage that *V*_ch_ could be systematically varied by changing the pore diameter, the internal dimensions and shape of the nanochannel in future studies, to further test our hypothesis and potentially refine the model.

For the 10 kbp and 48.5 kbp DNA, we moreover observed that after continued operation, DNA translocation eventually ceased and that the nanopipette reached a “blocked” state. This was accompanied by a marked drop of the current in the DC channel (albeit not to zero) and a significant increase of the electric noise in the AC channel. Notably, the transitions between the open, crowded, and blocked states were found to be reversible, which inspired a proof-of-concept experiment exploiting the crowding effect for sample processing and incubation. For this purpose, we filled the nanopipette with a solution containing streptavidin-modified, 40 nm gold nanoparticles, translocated biotin-functionalized DNA into the nanopipette, and after 20 min incubation, reverse translocated the DNA out of the pipet. Analysis of the translocation data allowed for the identification of various types of events associated with bare DNA, DNA-nanoparticle complexes, as well as seemingly simultaneous translocation of DNA and nanoparticles (“co-translocation”). Importantly, we were able to demonstrate through detailed analysis of the event substructure that the nanoparticles can be bound specifically to the DNA and that target capture has indeed been successful. This integration of sample incubation and detection into the nanopipette may therefore represent a bioanalytical paradigm, which could be extended to other targets such as proteins or RNA and different electrolytic media. The latter could include asymmetric configurations, i.e., with different solutions on the in- and outside of the nanopipette, adding substantial flexibility and generality to this analytical approach.

## Methods

Ag/AgCl electrodes were freshly prepared using anodization as previously described.^20^ First, 10 cm of silver wire (0.25 mm diameter, 99.99% purity, Goodfellow) was cut and immersed in 38% v/v nitric acid (Sigma) for 10 s, then washed with Milli-Q water (18 MΩ, Merck Millipore) to remove surface impurities. The cleaned wires were soldered to gold contact pins and submerged in the 4 M LiCl 1xTE solution. Anodization was performed in an electrochemical cell using a gold wire (99.99% purity, Goodfellow) as a counter electrode and applying a current of 1 mA until the electrode surface turned black/purple.

Nanopipettes were fabricated using laser mechanical pulling of plasma-cleaned filamented quartz capillaries (outer diameter: 1 mm, inner diameter: 0.5 mm, length: 7.5 cm, Sutter Instruments, Novato) with a P2000 pipet puller. The pulling parameters were optimized using two lines: line 1 (heat: 700, filament: 5, velocity: 35, delay: 150, pull: 75) and line 2 (heat: 700, filament: 0, velocity: 15, delay: 128, pull: 200). To mitigate thermally induced inconsistencies in pore size, the room temperature was maintained at approximately 20 °C. The taper length of the nanopipettes was subsequently determined using optical microscopy (Nikon, T-105) and ranged from 3.0 to 3.2 mm in the present study, cf. Section S6.

The pore diameter *d*_p_ was determined from the slope of the *I*/*V* curve measured in 4 M LiCl electrolyte solution using a custom-designed cell, based on eq 1(23)
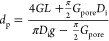
1where *G*_pore_ is the conductance of the pore, *l* is the taper length of the nanopipette (by optical microscopy), *D*_i_ is the inner diameter of the capillary (0.5 mm, as per manufacturer), and *g* is the conductivity of the electrolyte solution (173 mS cm^–1^).^20^ Values obtained for *d*_p_ were between 12 and 23 nm in this study, cf. Section S6.

To prevent or reduce the formation of air bubbles, the electrolyte solution was slowly filled back and forth into the nanopipette using a Microfil needle. Only nanopipettes displaying an Ohmic current–voltage (*I*/*V*) relationship were used, while those that did not meet this criterion were discarded. Ohmic behavior was defined using the ion current rectification ratio (*r*), calculated as *r* = |*I*–/*I*+|,^34^ at ±0.5 V. Nanopipettes with r values between 0.9 and 1.2 were considered acceptable.^34^

For DNA translocation experiments: 4,7,10 (Thermo Fisher, NoLimits DNA), 48.5 (NEB) and biotinylated 10 kbp DNA PCR product (detailed synthesis in the Section S1) were injected separately to the bulk solution in a custom designed liquid cell containing 0.1–3 mL of 4 M LiCl (bulk DNA concentrations 40–600 pM, as indicated). The liquid cell was housed in a double Faraday cage to reduce electric interference. A negative bias value means that the electrode outside the nanopipette is biased negatively, thereby resulting in an electrophoretic driving force for (negatively charged) DNA to translocate into the pipet.

Experiments were conducted in a semiautomated fashion using in-house MATLAB code with a sequence of applied biases, where for each bias, 100 data files of 10 s each were collected before the next bias value was applied. Under the electrolyte conditions used, typical DNA translocation leads to a decrease of pore current and events with negative polarity. However, for convenience, we use the absolute event magnitudes throughout this work.

Data recording was performed at a sampling rate of 1 MHz using a custom-built low-noise, high-bandwidth amplifier connected to the digital oscilloscope for analog-to-digital conversion (Picoscope 4262, Pico Technology), as reported previously.^5,6^ Briefly, in this design, the input current is split into two output channels, namely the “DC” and “AC” channels. The former contains slow modulations of the input current (cutoff frequency ∼ 7 Hz), including the steady-state pore current, which may be used to estimate *G*_pore_. The AC channel contains fast modulations of the input current, for example, (short-lived) translocation events, and is normally zero mean, facilitating baseline correction. For the results presented here, the AC channel output was filtered using an eight-pole low-pass (analog) Bessel filter (cutoff: 100 kHz, Krohn-Hite Corporation) and, in some cases, also digitally filtered as indicated.

### Event Detection

Data analysis involved zero-order baseline background correction of the AC channel output to remove minor current offsets (<10 pA), when necessary. Using custom-built MATLAB code, events were detected with a 5σ threshold, unless specified otherwise, where σ was the standard deviation of the AC channel noise. For each detected event, relevant segments of the current–time trace were extracted from and up to the adjacent zero crossings and relevant event characteristics were determined, such as the event current magnitude, Δ*I*_max_ and the effective event current, Δ*I*_e_ (from the event charge and its duration). Additional characteristics included the event duration based on 1σ threshold crossings (τ), which we found to better capture the characteristics of events with complex shapes, or the event duration at full-width half-maximum.^5,20^ For subevent detection, a separate threshold search was performed on each event with a threshold value of 200 pA, relative to the median baseline of the event (calculated between 0.1 and 0.9 of the relative event duration), and the number, position, and other subevent characteristics extracted.

The separation of linear and folded event populations was conducted by first extracting the overall DNA event cluster in the Δ*I*_eff_ or Δ*I*_max_ vs τ scatter plot using DBSCAN. For these events, five features, namely the event duration (τ), the effective current (ΔI_e_), the maximum event current (Δ*I*_max_), the AC channel noise, and the event charge (q), were first standardized (z-score) and then subjected to Principal Component Analysis (PCA). The first two principal components were retained and subsequently separated using k-means clustering with two populations. The cluster with the longer τ_m_, as determined from a Gaussian fit of the respective τ distribution, was associated with linear DNA translocation events and color-coded in the corresponding Δ*I*_e_ or Δ*I*_max_ vs τ plots, as indicated. Further information can be found in of the Section S4.

## Supporting Information Available

DNA preparation protocols, gel electrophoresis data, additional translocation data not included in the main manuscript and representative examples of translocation events for the DNA/nanoparticle experiment shown in Figure 6 (PDF)

## Notes

A preprint of this work is available: Rand A. Al-Waqfi; Cengiz Khan; Oliver J. Irving; Lauren Matthews; Tim Albrecht, Crowding Effects during DNA Translocation in Nanopipettes. 2025, arXiv:2501.14347v2. arXiv. 10.48550/arXiv.2501.14347 (accessed April 13th, 2025)
