# Supplementary material for: Crowding Effects during DNA Translocation in Nanopipettes
Source: ACS Nano. 2025 Apr 23;19(17):16803–12. doi: 10.1021/acsnano.5c01529 (PMC12060640; doi:10.1021/acsnano.5c01529)
Supplement: Supplementary file 1 — nn5c01529_si_001.pdf [file nn5c01529_si_001.pdf]

Crowding Effects during DNA Translocation in Nanopipettes

Rand A. Al-Waqfi[$,£], *Cengiz J. Khan*[$], *Oliver J. Irving*[$], *Lauren Matthews*[$,&], *Tim Albrecht*[$]

[$] University of Birmingham, School of Chemistry, Edgbaston Campus, Birmingham B15 2TT, United Kingdom

[£] Department of Medicinal Chemistry and Pharmacognosy, Faculty of Pharmacy, Jordan University of Science and Technology, P.O. Box 3030, Irbid 22110, Jordan

[&] Federal Institute for Materials Research and Testing, Department 6, Unter den Eichen 87, 12205 Berlin, Germany

Supporting Information

1. **Synthesis of 10 kbp biotin-functionalised DNA:**

Synthesis of 5'biotin-functionalised DNA was conducted using polymerase chain reaction (PCR). The forward (5'-TCATCAGGGCGAGATGCTCAATG) and reverse primer (5'-[biotin]-AAGGCGTTTCCGTTC TTCTTCGT) were provided by Integrated DNA Technologies (IDT) and reconstituted to a concentration of 10 μM in nuclease-free water. Briefly, the reaction was carried out using 25 μL of Q5 High-Fidelity 2X Master Mix (NEB), 2.5 μL of each primer, 1 μL of λ DNA as template DNA (1 ng/μL, NEB) and 19 μL NFW (VWR international). The reaction components were gently mixed by pipetting and briefly centrifuged to ensure homogeneity. LoBind© tube was placed inside PrimeG© thermocycler (Cole Palmer) and thermal cycling conductions were applied as follows: an initial denaturation at 98˚C for 30 seconds, followed by 30 cycles of 98˚C for 10 seconds, 70˚C for 30 seconds, and 72˚C for 5 minutes, concluding with a final extension at 72˚C for 2 minutes. PCR product was purified using Monarch PCR and DNA Clean-Up kit (NEB) and eluted in nuclease free water. The integrity and expected size of the biotinylated DNA fragment were verified by 1% TAE agarose gel electrophoresis and quantified using a UV-Vis spectrophotometer.

2. Gel Electrophoresis results for DNA standards and 10 kbp PCR product:

4 kbp, 7 kbp and 10 kbp DNA samples (NoLimits DNA fragments, Thermo Fisher) as well as 48.5 kbp DNA (NEB) were used. The purity of DNA standards and the success of the above-mentioned PCR reaction were confirmed by gel electrophoresis. In figure S1A, lanes 2-4 showing bands corresponding to 4 kbp, 7 kbp and 10 kbp DNA, respectively.

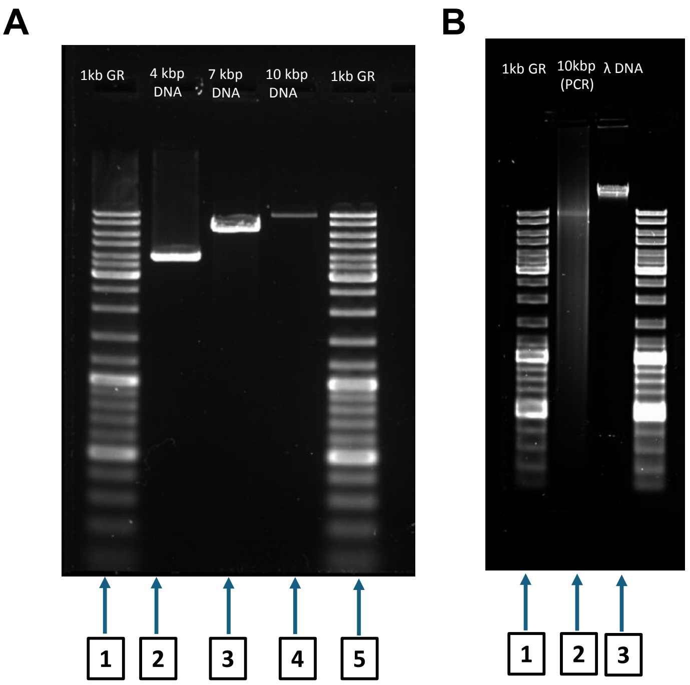

Figure S1: Gel electrophoresis results for: A) 4, 7 and 10 kbp DNA samples (lanes 2-4). B) biotin-functionalised 10 kbp PCR product (lane 2) and 48.5 kbp DNA fragment (lane 3). The gels were run for 49 min at 80 V (1% (w/v) agarose gel prepared in 1× TAE buffer).

3. Examples of current-time traces (AC channel) with typical translocation events

The traces below are example outputs for 4 kbp DNA from the AC channel (sampling time: 1 μs, low-pass filtered at 100 kHz), see Methods section in the main text. The transient on the left of each measurement results from switching V_{bias} to the specified value. Data in panel A were recorded at V_{bias} = +0.8 V while no DNA was inside the nanopipette and therefore no events observed. The measurement in panel B was recorded at V_{bias} = -0.6 V, resulting in DNA translocation into the nanopipette. Insets: example events, likely showing the translocation of folded and linear DNA, respectively. Finally, data shown in panel C were measured at V_{bias} = +0.6 V. Translocation events are now in the upwards direction, but note that the total current is composed of both AC and DC channels. Thus, under the high-ionic strength conditions used (4 M LiCl), DNA translocation events are decreases in the overall current (blockages).

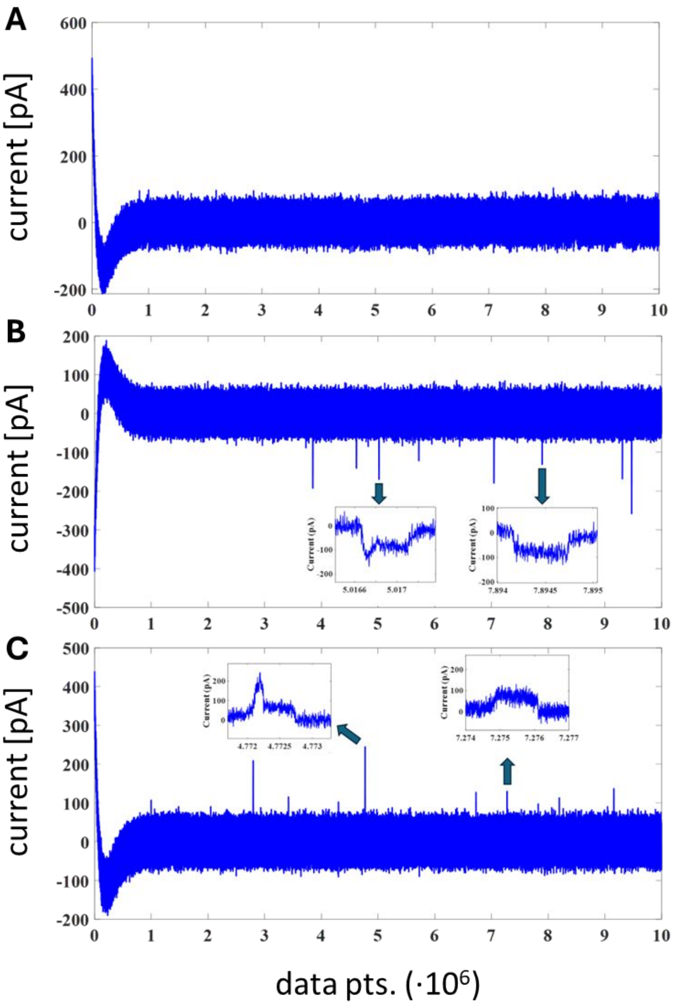

Figure S2: Raw data output for 4 kbp DNA (AC channel), current vs. data points. A) V_{bias} = +0.8 V, no DNA translocation occurs as the initial DNA concentration inside the pipette is zero. Panels B/C): V_{bias} = -0.6 V and +0.6 V. DNA events are observed in both cases.

4. Separation of linear and folded DNA translocation events – an example

Following the description of the separation of linear and folded DNA translocation events in the main manuscript (cf. Methods), we further illustrate this process here. To this end, fig. S2 (left) shows the ΔI_{eff} vs. τ scatter plot from a translocation experiment with 4 kbp DNA (V_{bias} = -0.8 V, 4 M LiCl). A distinct cluster of DNA translocation events is visible and indicated by the ellipse. This subset of events is first extracted using DBSCAN (or another suitable clustering method). After z-score standardisation, PCA analysis is performed based on an

expanded set of five features (see main manuscript), the first two principal components retained and subsequently clustered into two sub-populations using k-means clustering. The sub-population formally associated with linear events is colour-coded in red. Two example events for linear and folded DNA translocation are shown on the right.

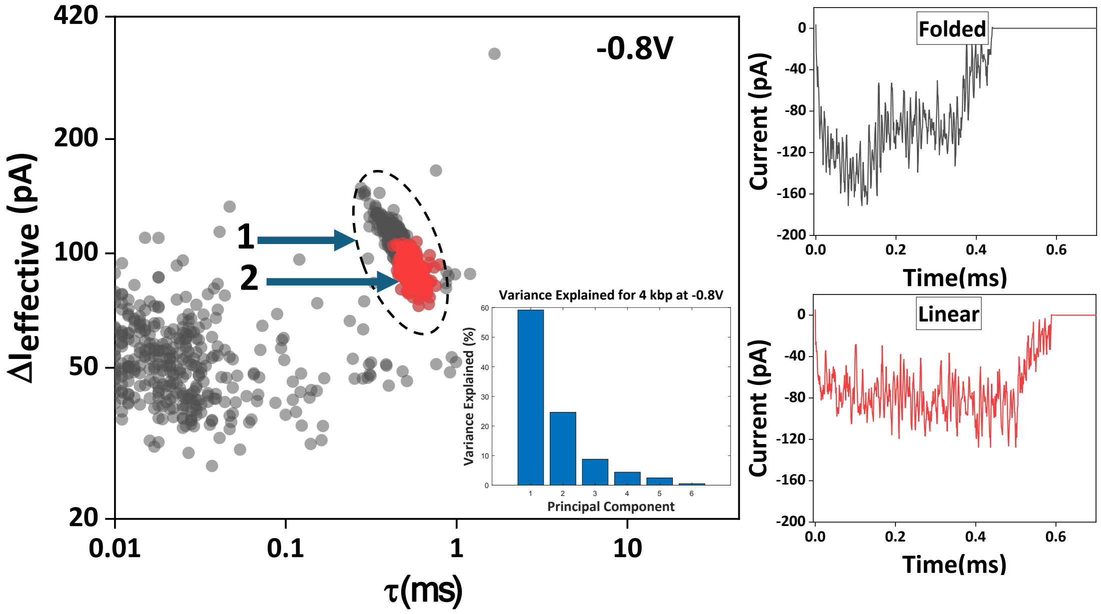

Figure S3. Left: scatter plot of ΔI_{eff} vs. τ for 4 kbp DNA ($c_{DNA,out}$ = 300 pM, -0.8 V). The overall DNA-related cluster is enclosed within an ellipse and shown in black. Linear translocation events, isolated in step 2 using PCA and k-means clustering, are highlighted in red. The Scree plot (bottom inset) illustrates the variance captured by each principal component, with the first two components explaining ~85% of the total variance. Right: two representative current-time events, namely folded (top, black) and linear (bottom, red).

5. Translocation data for other DNA lengths, ΔI_{eff} vs. τ scatter plots (examples)

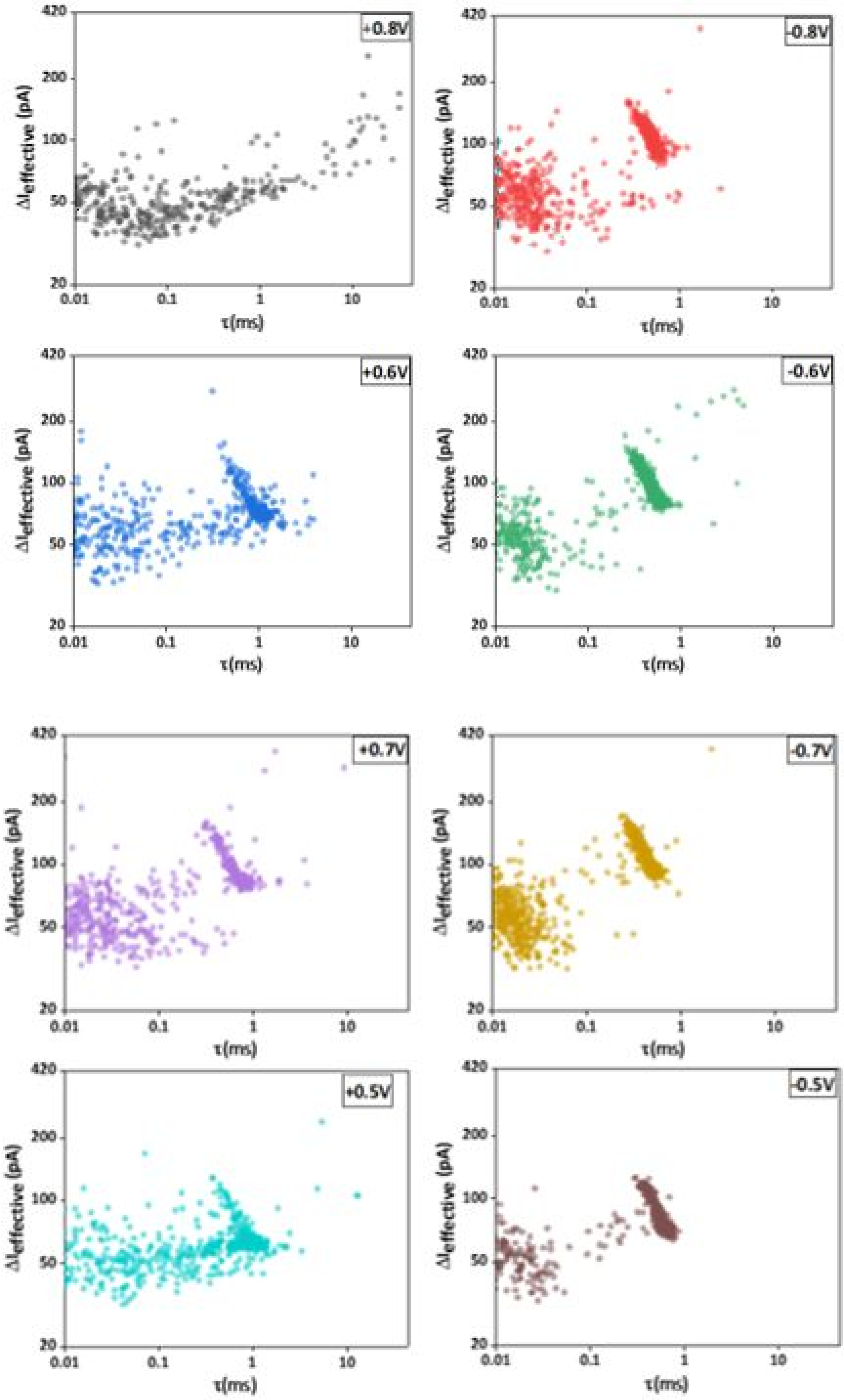

Figure S4: Scatter plots of ΔI_{eff} vs. τ for 4 kbp DNA ($c_{DNA,out}$ = 300 pM), bias applied in sequence from left to right. No DNA cluster at +0.8 V, since $c_{DNA,in}$ = 0 at the beginning of the experiment. The applied bias sequence was from left to right, top to bottom.

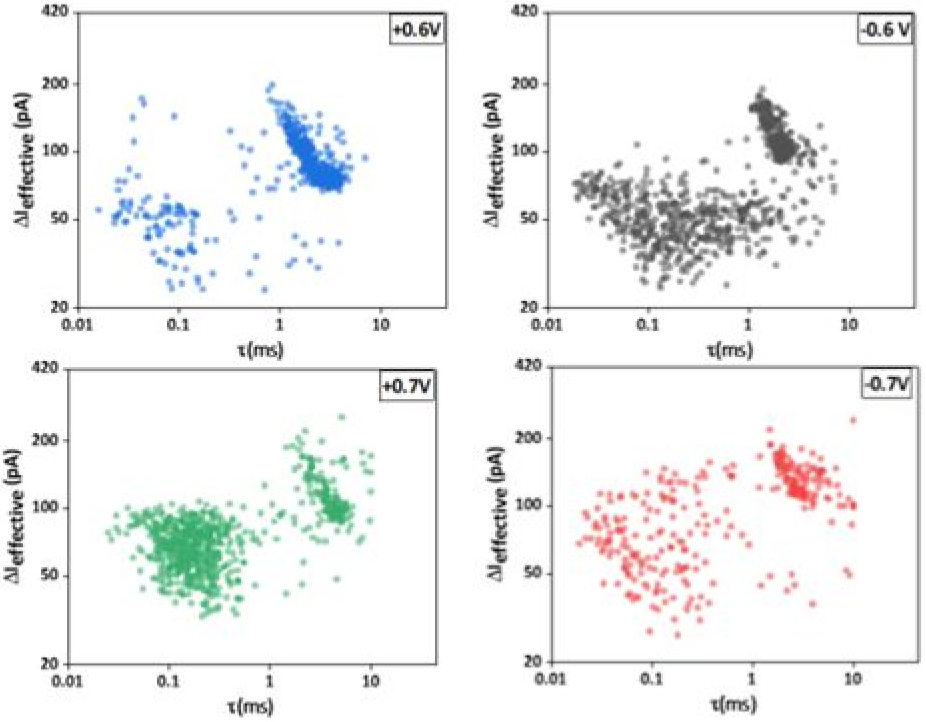

Figure S5: Scatter plots of ΔI_{eff} vs. τ for 7 kbp ($c_{DNA,out}$ = 600 pM). The applied bias sequence was from left to right, top to bottom.

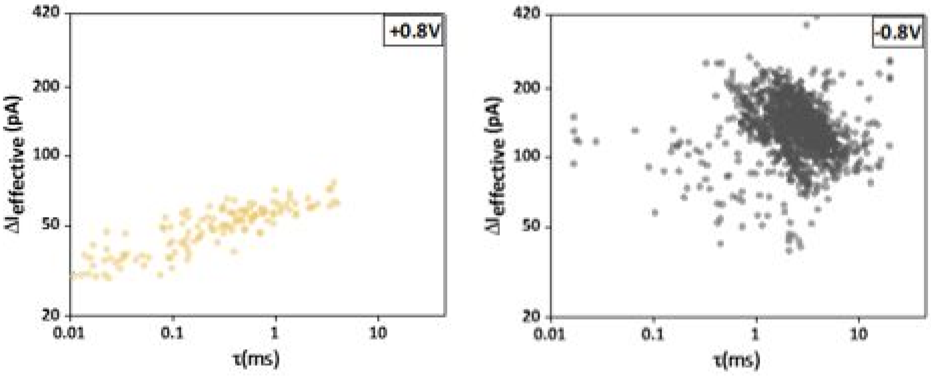

Figure S6: Scatter plots of of ΔI_{eff} vs. τ for 10 kbp DNA ($c_{DNA,out}$ = 600 pM).

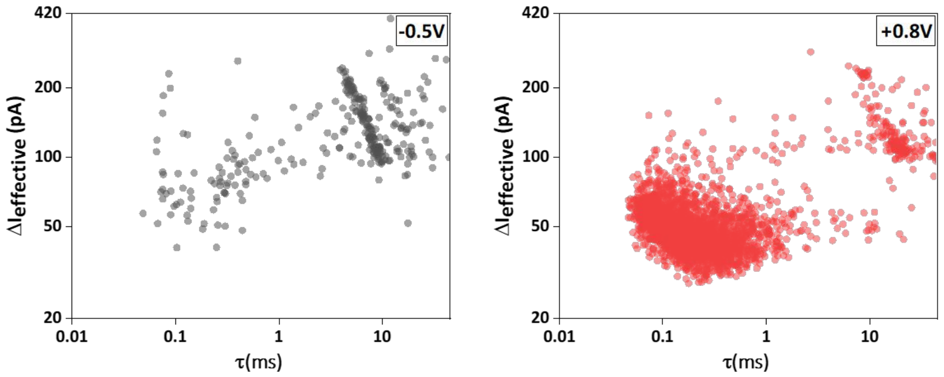

Figure S7: Scatter plots of ΔI_{eff} vs. τ for 48.5 kbp λ-DNA ($c_{DNA,out}$ = 300 pM). Data were filtered at 10kHz.

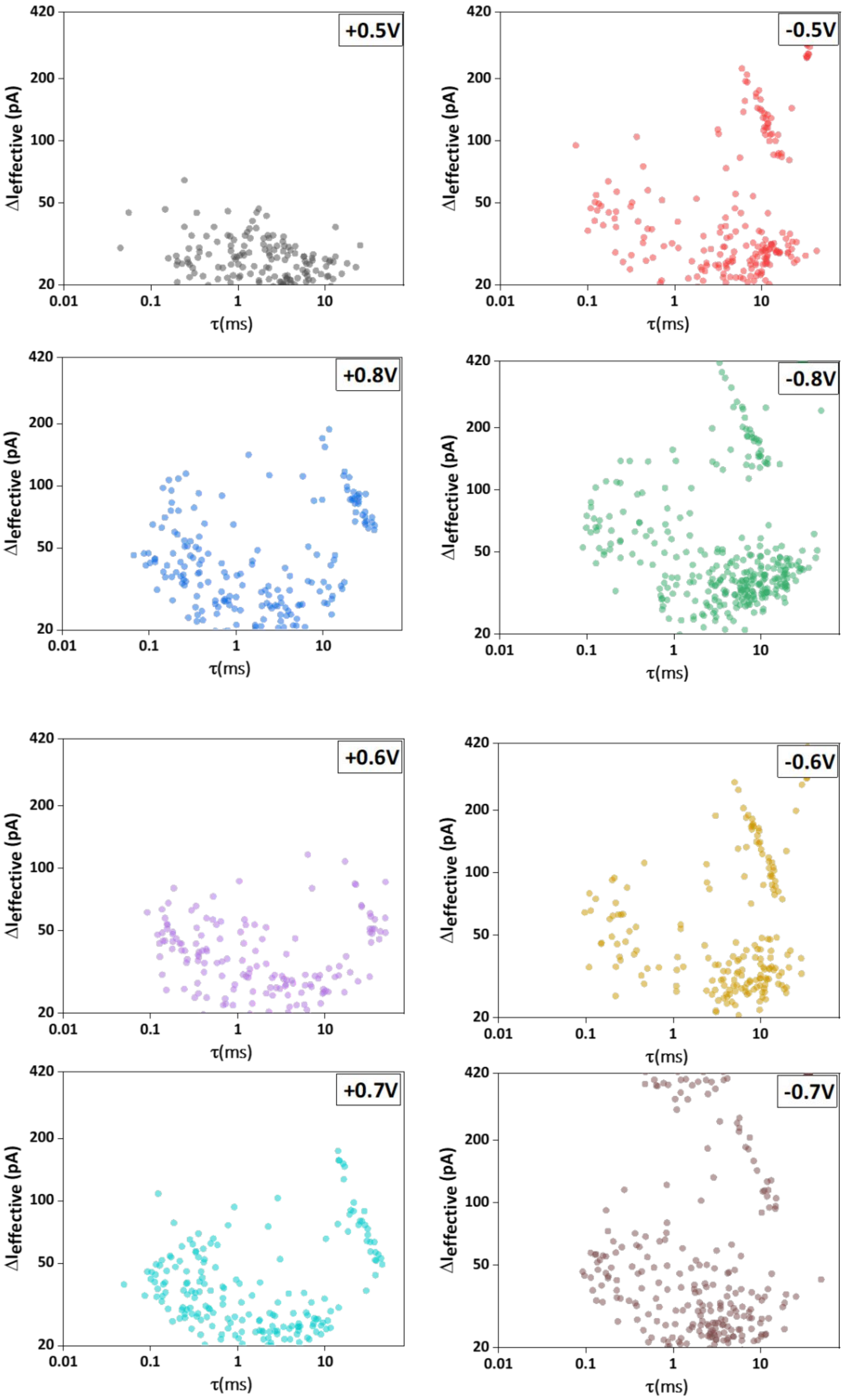

Figure S8: Scatter plots of ΔI_{eff} vs. τ for λ DNA ($c_{DNA,out}$ = 40 pM). The applied bias sequence was from left to right, top to bottom.

6. Nanopipette characterisation: physical dimensions and conductance

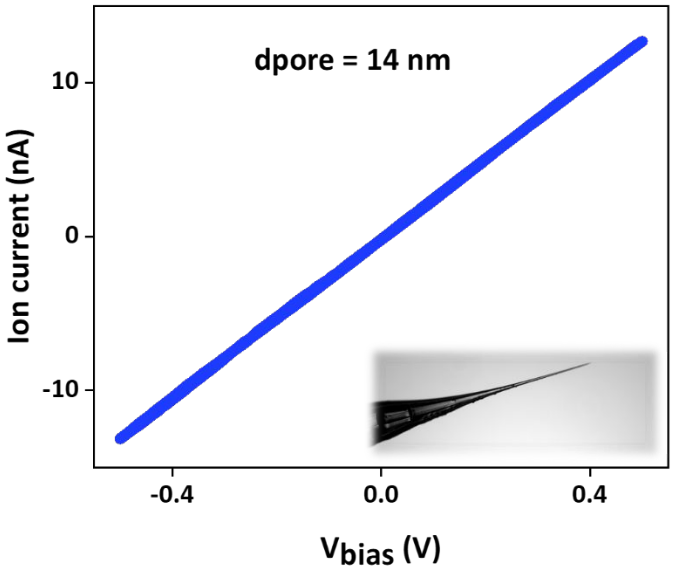

Figure S9: I/V_{bias} curve for a typical quartz nanopipette in 4 M LiCl with 10 mM TE buffer. The conductance is G = 27.5 nS, as determined from the average of the slopes from the forward and reverse scan between ±0.5 V. Bottom inset: optical micrograph showing the taper of the same pipette, with a length of approximately 3.17 mm

Table S1: Nanopipette characterisation and DNA concentrations

	**4 kbp**	**4 kbp**	**7 kbp**	**10 kbp**	**48.5 kbp**	**48.5 kbp**
**Est. Pore size**	14 nm	20 nm	14 nm	18 nm	12 nm	23 nm
**Taper length**	3.12 mm	3.05 mm	3.17 mm	3.2 mm	3.132 mm	3.1 mm
**Conductance**	26.76 nS	48.4 nS	27.5 nS	36.9 nS	31.6 nS	44.1 nS
**DNA conc.**	300 pM	300 pM	600 pM	600 pM	300 pM	40 pM

7. Estimate of V_{ch} based on a worm-like chain model for DNA

In order to estimate the volume of the nanoconfined region in the nanopipette tip, V_{ch}, we assume that $N_{DNA} \cdot V_{DNA} = V_{ch}$ at the transition from phase 1 to 2 during translocation experiments (i.e., that the space is completely filled, albeit not closely compacted). For the description of the DNA, we adopted the worm-like chain model which yields for the mean-square radius of gyration, R_g:[1]

$$\langle R_g^2 \rangle = \frac{b^2 N_{Kuhn}}{6}$$

b is the Kuhn length and N_{Kuhn} the number of Kuhn monomers in the chain. N_{Kuhn} is equal to $\frac{N_{bp} \cdot d_{bp}}{b}$ where N_{bp} is the number of base pairs in the strand and d_{bp} the average distance between two adjacent ones. Noting that b is equal to two times the persistence length P, we have:

$$\langle R_g^2 \rangle = \frac{2P \cdot N_{bp} \cdot d_{bp}}{6}$$

Finally, since $\frac{R_H}{R_G} = \frac{3}{8}\sqrt{\pi} \approx 0.665$, we can write an expression for the spherical volume of a DNA strand in terms of the hydrodynamic radius R_H as $V_{DNA} = \frac{4}{3}\pi \cdot R_H^3$.

Since $N_{DNA} = V_{ch}/V_{DNA}$, see main text, we obtain:

$$N_{DNA} = \frac{V_{ch}}{V_{DNA}} = \frac{3V_{ch}}{4\pi\left(\frac{3}{8}\sqrt{\pi} \cdot R_G\right)^3} \approx 4.22 \cdot \frac{V_{ch}}{\left(P \cdot d_{bp}\right)} \cdot (N_{bp})^{-\frac{3}{2}}$$

Hence, a plot of N_{DNA} vs. $N_{bp}^{-\frac{3}{2}}$ should show a linear correlation with slope $4.22 \cdot \frac{V_{ch}}{(P \cdot d_{bp})}$, cf. fig. 4C, which based on known values of P and d_{bp} allows for the estimation of V_{ch}.

8. Additional translocation statistics for the DNA samples studied in this work

Table S2:

DNA length (kbp)	V_{bias} (V)	ratio, linear vs. folded	τ_m (linear events) (s)	G_{pore} (nS)	no. of events
4	-0.6	0.596	0.000538	48.4	1028
4	-0.5	0.604	0.000596	48.4	1450
4	-0.7	0.569	0.000480	48.4	1778
4	-0.8	0.562	0.000442	48.4	1077
4	-0.4	0.509	0.000571	48.4	432
4	0.6	0.6348	0.000816	48.4	419
4	0.5	0.549	0.000686	48.4	271
4	0.7	0.6729	0.000837	48.4	691
4	-0.5	0.629	0.000615	26.76	383
4	-0.8	0.55	0.000551	26.76	673
4	-0.6	0.59055	0.000568	26.76	464
4	-0.7	0.59666	0.000486	26.76	534
4	-0.4	0.643	0.000723	26.76	253
4	0.6	0.7703	0.001060	26.76	283
4	0.7	0.834	0.000791	26.76	362
4	0.5	0.8	0.001070	26.76	280
7	-0.5	0.576	0.001600	27.5	520
7	-0.8	0.538	0.001500	27.5	1069
7	-0.6	0.556	0.002300	27.5	254
7	-0.7	0.42	0.004000	27.5	193
7	0.5	0.50993	0.006500	27.5	183
7	0.8	0.6319	0.002000	27.5	398
7	0.6	0.61966	0.003000	27.5	573
7	0.7	0.4918	0.004600	27.5	151
10	-0.8	0.4972	0.004040	36.9	1215
10	-0.6	0.572	0.001640	36.9	131

10	-0.7	0.462	0.002970	36.9	1051
10	0.7	0.552	0.003960	36.9	1035
48.5	-0.5	0.483	0.008590	31.6	172
48.5	0.8	0.5703	0.021520	31.6	154

9. Loading, blocking and unloading of nanopipettes: 10 kbp DNA

Translocation of 10 kbp DNA occurred in different phases, from uninhibited translocation (phase 1) to delayed translocation (phase 2), fig. S9 panels A + B, and ultimately blockage (phase 3). Compared to 48.5 kbp DNA, the latter required a larger number of DNA translocation events, but as in the case of the longer DNA, G_{pore} drops and AC channel noise increase significantly. Note that this effect is reversible under the experimental conditions used, as demonstrated by the bias reversal (and unloading) to V_{bias} = +0.7 V, panel C.

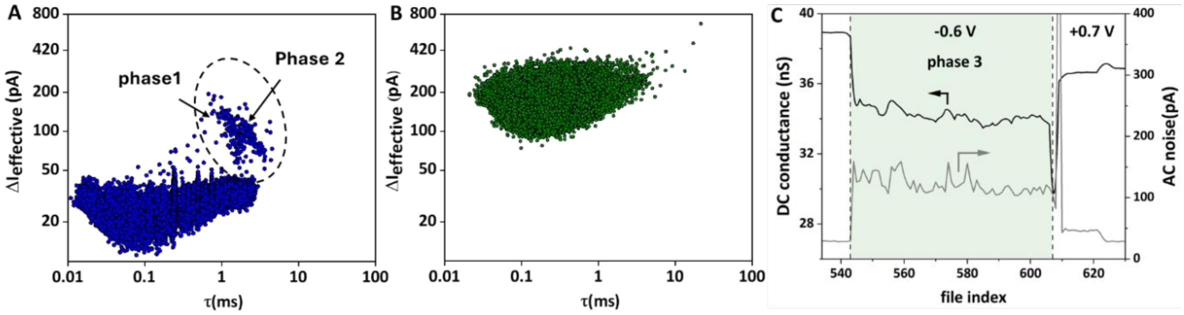

Figure S10*:* A/B) Scatter plots of ΔI_{eff} vs. τ for 10 kbp DNA, for the phases 1 + 2 (A) and phase 3 (B) (V_{bias} = -0.6 V, event detection threshold of 1.5σ). C) G_{pore} and standard deviation of the current noise in the AC channel, as a function of file index (1 file = 10 s run time). Phase 3 is highlighted in green. The final set at V_{bias} = +0.7 V shows the reversal of the blocking, with a recovery of G_{pore} and current noise after DNA is transported out of the nanopipette.

10. DNA/nanoparticle translocation: example events with sub-event structure

While the event threshold was at 5σ, where σ is the standard deviation of the current noise in the AC channel (see Methods), events below are plotted from the last zero crossing before event (left-hand side) to first zero recrossing after the event (sharp drop, right-hand side). The threshold for sub-event detection was 200 pA, relative to the median of the event baseline between 0.1 and 0.9 relative event time.

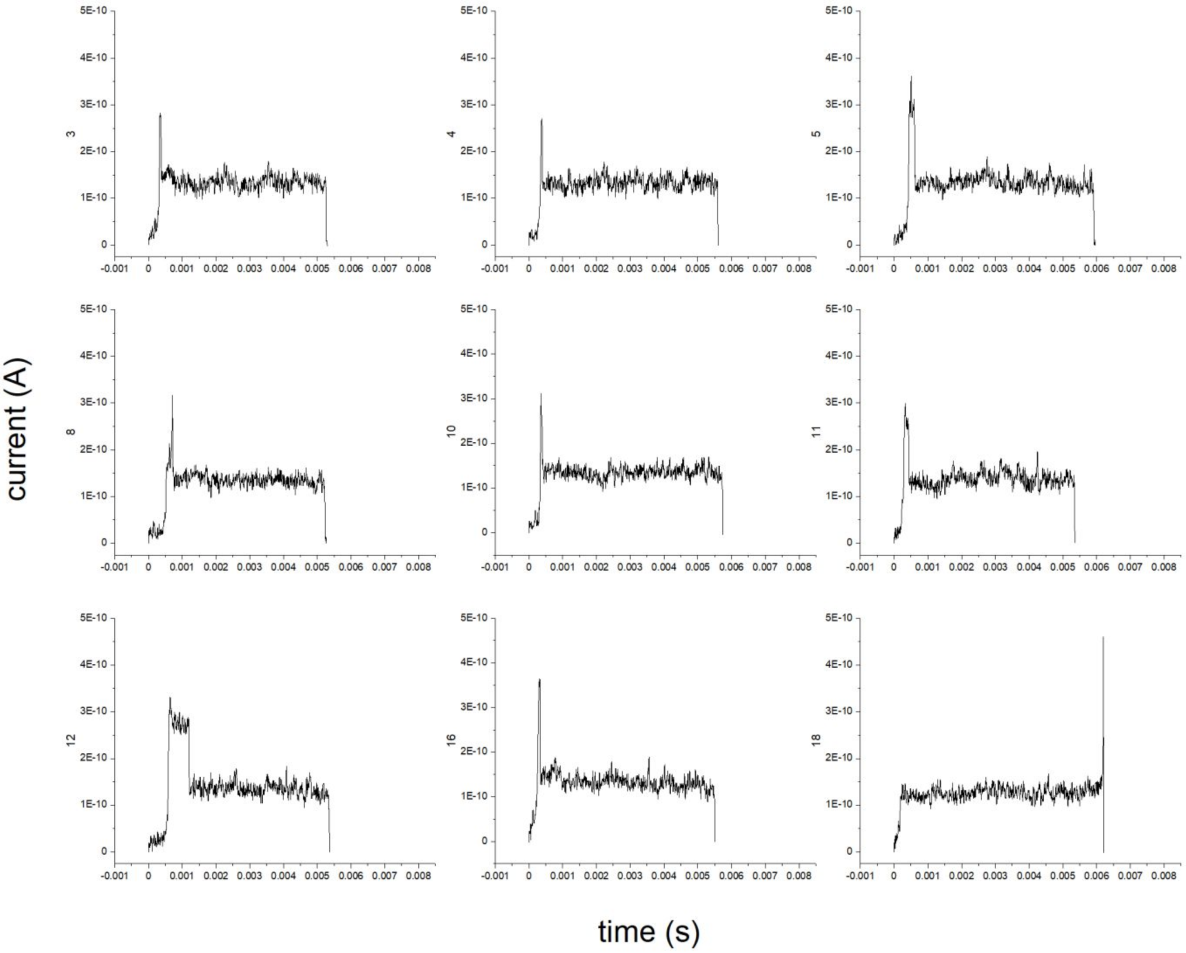

Figure S11: Current-time traces for the first 9 events formally identified with **one** sub-event. The current range is from -25 pA to 500 pA for all events.

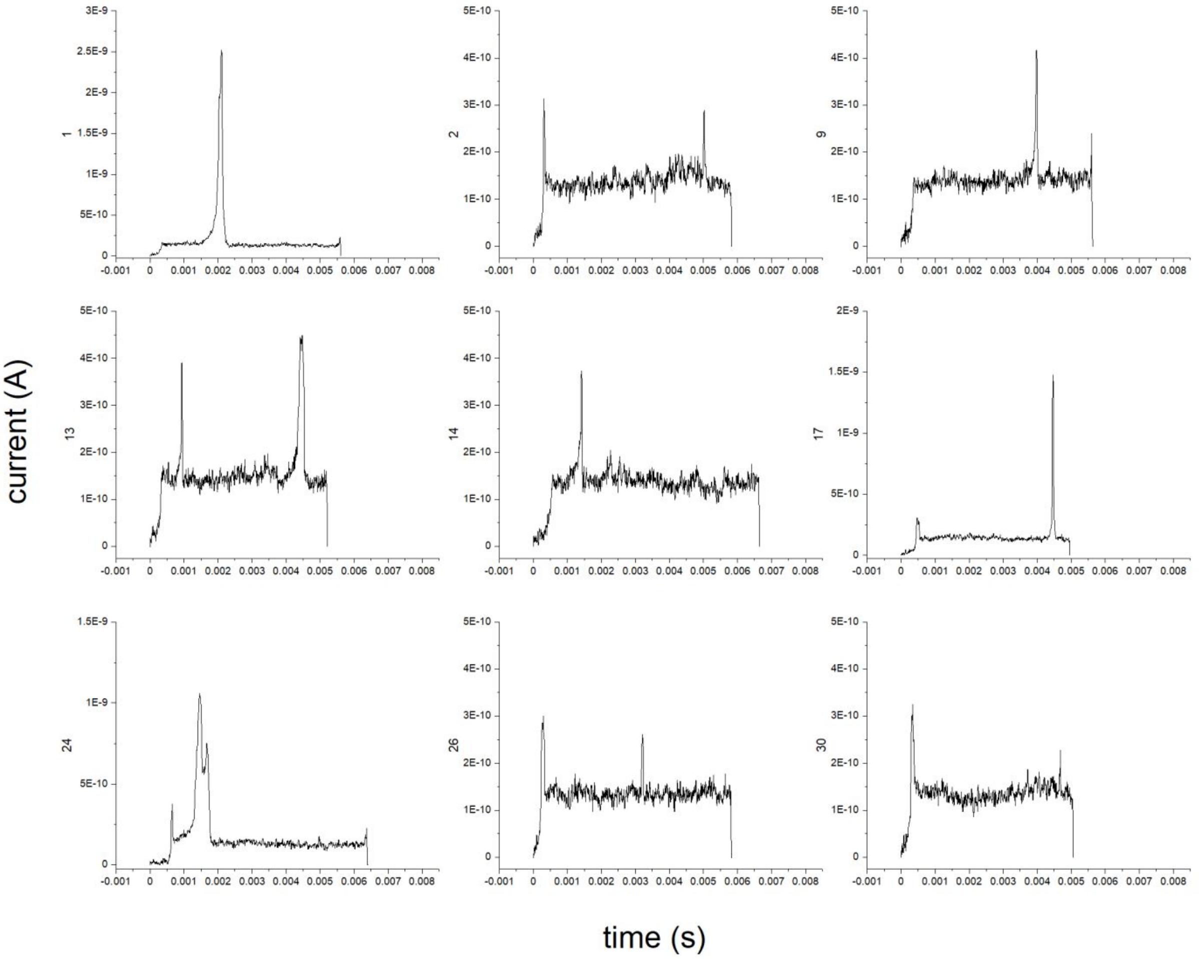

Figure S12: Current-time traces for the first 9 events formally identified with **two** sub-events. The current range is from -25 pA to 500 pA for all events, except events “1”, “17” and “24”, which have been re-scaled to an upper limit of 3 nA, 2 nA and 1.5 nA to improve visibility across the set.

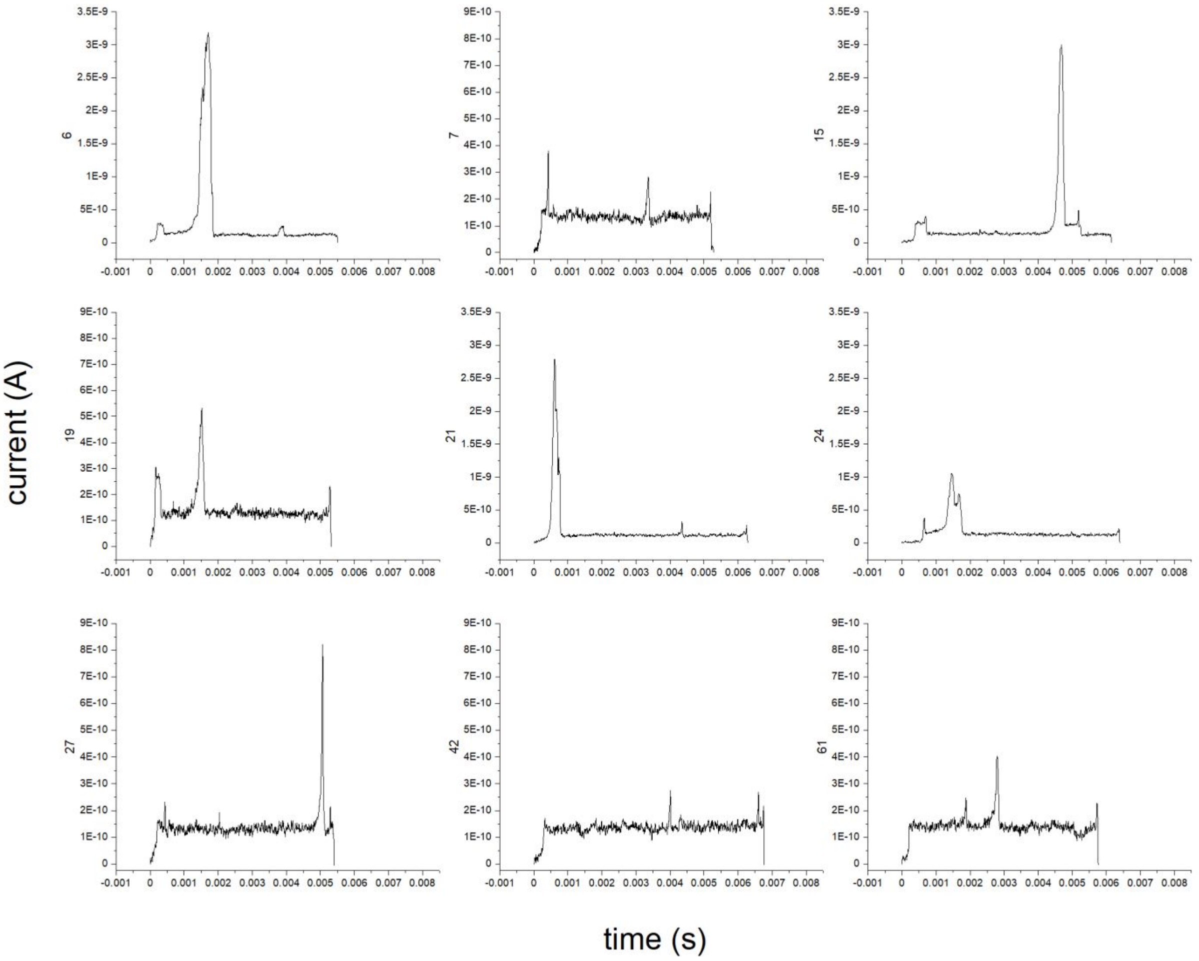

Figure S13: Current-time traces for the first 9 events formally identified with **three** sub-events. The current range is from -25 pA to 3.5 nA (events 6, 15, 21, 24) or -25 pA to 0.9 nA (all others).

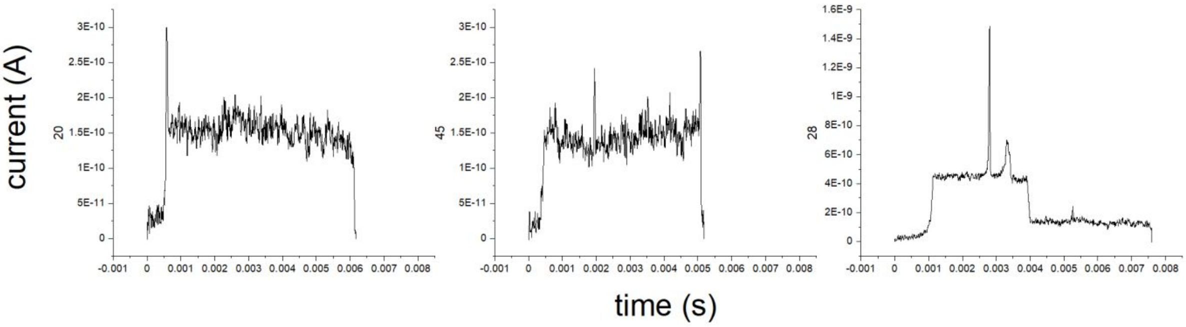

Figure S14: Current-time traces for three events that were formally identified with **4 or more** sub-events, the current range is from -25 pA to 325 pA (events 20 and 45, formally 4 sub-events) and from -100 pA to 1.6 nA (event 28, formally 12 sub-events). From visual inspection, these appear to be at least in part misclassifications, for example due to the underestimation of the event baseline level (events 20, 45) or the complex shape of the event (event 28).

References:

1. I. Teraoka, "Polymer Solutions - An Introduction to Physical Properties", Wiley & Sons (2002), chapter 3, p. 186
